# A Standardized Extract Prepared from Red Orange and Lemon Wastes Blocks High-Fat Diet-Induced Hyperglycemia and Hyperlipidemia in Mice

**DOI:** 10.3390/molecules26144291

**Published:** 2021-07-15

**Authors:** Santina Chiechio, Magda Zammataro, Massimo Barresi, Margherita Amenta, Gabriele Ballistreri, Simona Fabroni, Paolo Rapisarda

**Affiliations:** 1 Section of Pharmacology, Department of Drug and Health Sciences, University of Catania, 95123 Catania, Italy; magzam@libero.it (M.Z.); barresi.massimo1970@gmail.com (M.B.); 2 Oasi Research Institute IRCCS, 94018 Troina, Italy; 3 Consiglio per la Ricerca in Agricoltura e l’Analisi dell’Economia Agraria, Centro di Ricerca Olivicoltura, Frutticoltura e Agrumicoltura, 95024 Acireale, Italy; margherita.amenta@crea.gov.it (M.A.); gabriele.ballistreri@crea.gov.it (G.B.); simona.fabroni@crea.gov.it (S.F.); paolo.rapisarda@crea.gov.it (P.R.)

**Keywords:** citrus fruits, phytoextract, hyperglycemia, hyperlipidemia, high-fat diet

## Abstract

Citrus fruits are a rich source of high-value bioactive compounds and their consumption has been associated with beneficial effects on human health. Red (blood) oranges (*Citrus sinensis* L. Osbeck) are particularly rich in anthocyanins (95% of which are represented by cyanidin-3-glucoside and cyanidin-3-6″-malonyl-glucoside), flavanones (hesperidin, narirutin, and didymin), and hydroxycinnamic acids (caffeic acid, coumaric acid, sinapic, and ferulic acid). Lemon fruit (*Citrus limon*) is also rich in flavanones (eriocitrin, hesperidin, and diosmin) and other polyphenols. All of these compounds are believed to play a very important role as dietary antioxidants due to their ability to scavenge free radicals. A standardized powder extract, red orange and lemon extract (RLE), was obtained by properly mixing anthocyanins and other polyphenols recovered from red orange processing waste with eriocitrin and other flavanones recovered from lemon peel by a patented extraction process. RLE was used for in vivo assays aimed at testing a potential beneficial effect on glucose and lipid metabolism. In vivo experiments performed on male CD1 mice fed with a high-fat diet showed that an 8-week treatment with RLE was able to induce a significant reduction in glucose, cholesterol and triglycerides levels in the blood, with positive effects on regulation of hyperglycemia and lipid metabolism, thus suggesting a potential use of this new phytoextract for nutraceutical purposes.

## 1. Introduction

Global production of citrus fruits is estimated at over 130 million tons per season, with oranges accounting for almost 70% of the total production, while lemons and limes account for about 15% [1]. In Italy, citrus processing factories process almost one million tons of citrus fruits every year (ISMEA, 2020), generating large amounts of waste (almost 60%) which, if not recycled, represents a huge amount of raw material to be disposed of, resulting in high costs. Citrus fruits contain numerous high-value bioactive compounds; their consumption has been associated with health-promoting effects in humans. Red (blood) oranges are particularly rich in anthocyanins (95% of which are cyanidin-3-glucoside and cyanidin-3-6″-malonyl-glucoside), flavanones (hesperidin, narirutin and didymin), and hydroxycinnamic acids (caffeic, coumaric, sinapic, and ferulic acids). All of these compounds provide health benefits as dietary antioxidants. The anthocyanin pigments in blood oranges are of particular interest as recent studies have shown their health benefits as dietary antioxidants due to their ability to scavenge free radicals [2]. Anthocyanins have also been associated with potential beneficial effects against various diseases, such as capillary fragility, diabetic retinopathy, and platelet aggregation in humans [3]. Recent studies have shown that the juice of ‘Moro’, a blood orange variety with the highest anthocyanin content, inhibits fat accumulation in mice fed with a high-fat diet [4]. Flavanones and hydroxycinnamic acids are currently used to treat capillary fragility and other diseases whose etiology is associated with the detrimental effects of free radicals [2,5,6]. Lemon fruit (*Citrus limon*) is also a rich source of bioactive compounds such as vitamin C, flavanones (eriocitrin, hesperidin, narirutin), hydroxycinnamic acids (ferulic, coumaric, sinapic), flavones (diosmetin 6,8-di-*C*-glucoside), and flavonols glycosides (rutin). Many studies have revealed its nutritional and health-promoting properties showing several biological functions such as antioxidant, anti-inflammatory, antiallergic, and antiviral activities [7,8,9,10,11,12].

The present work was aimed at investigating a potential beneficial effect of red orange and lemon extract (RLE), a standardized powder containing anthocyanins, polyphenols, eriocitrin, and other flavanones, obtained from the processing waste of blood oranges and lemons by a patented extraction method, on glucose and lipid metabolism in mice fed with a high-fat diet.

## 2. Results

### 2.1. Chemical Composition of RLE

The RLE dry powdered extract is standardized to contain ≥2.5% total anthocyanins, ≥15.0% total flavanones, and ≥1.5% total hydroxycinnamic acids. RLE used for the in vivo assays was analyzed by HPLC-MS. The results of individual anthocyanins, flavanones, and hydroxycinnamic acids identification and quantification are shown in Table 1, Table 2 and Table 3.

### 2.2. In Vivo Studies

High-fat diets (HFD) in mice are known to elicit a number of metabolic complications observed in humans, such as hyperglycemia and hyperlipidemia, by increasing blood levels of glucose, triglyceride, and cholesterol, leading to a huge increase in cardiovascular risk factors. Therefore, HFD in mice is a validated model to test compounds with potential protective effects against HFD-induced metabolic disorders [13].

**Figure 1:**
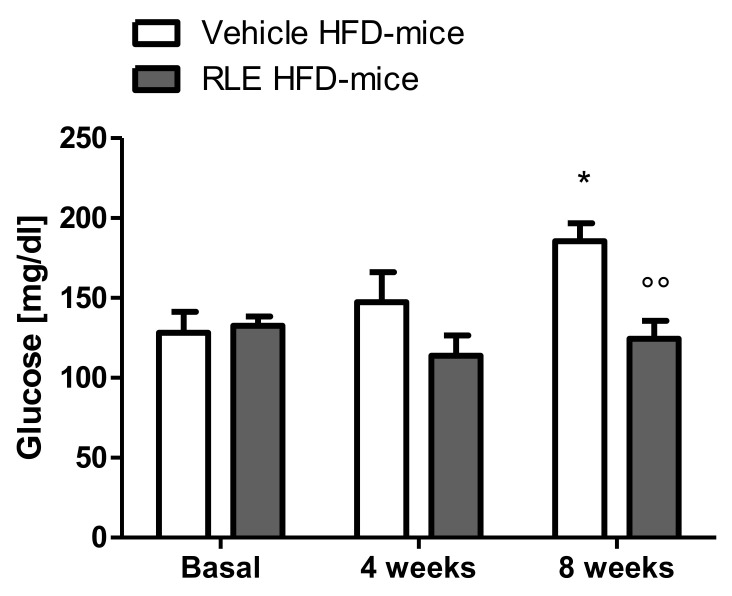
Eight weeks of RLE diet supplementation reduce glucose blood concentration in HFD-mice. RLE diet supplementation was able to block blood glucose level increase in CD1 mice fed on a high-fat diet (HFD-mice) after eight weeks of treatment. No significant differences were observed after four weeks of treatment with RLE. Data are means ± SEM of 9 to 12 mice per group. Two-way ANOVA followed by Bonferroni post-test. * *p* < 0.5 vs vehicle HFD-mice basal group; °° *p* < 0.1 vs vehicle HFD-mice 8 weeks group.

**Figure 2:**
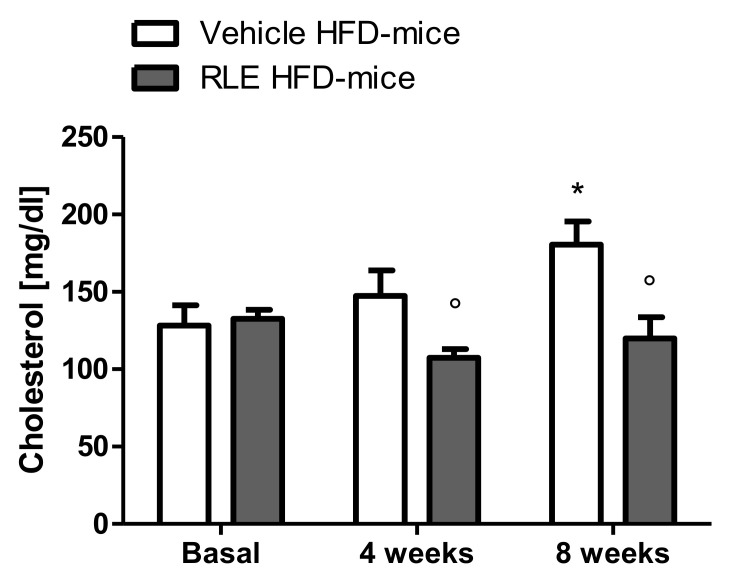
Eight weeks of RLE diet supplementation reduce cholesterol blood concentration in HFD-mice. RLE diet supplementation was able to block blood cholesterol increase in CD1 mice fed on a high-fat diet (HFD-mice) after four and eight weeks of treatment. Data are means ± SEM of 9 to 12 mice per group. Two-way ANOVA followed by Bonferroni post-test. * *p* < 0.5 vs. vehicle HFD-mice basal group; ° *p* < 0.5 vs vehicle HFD-mice 8 weeks group.

**Figure 3:**
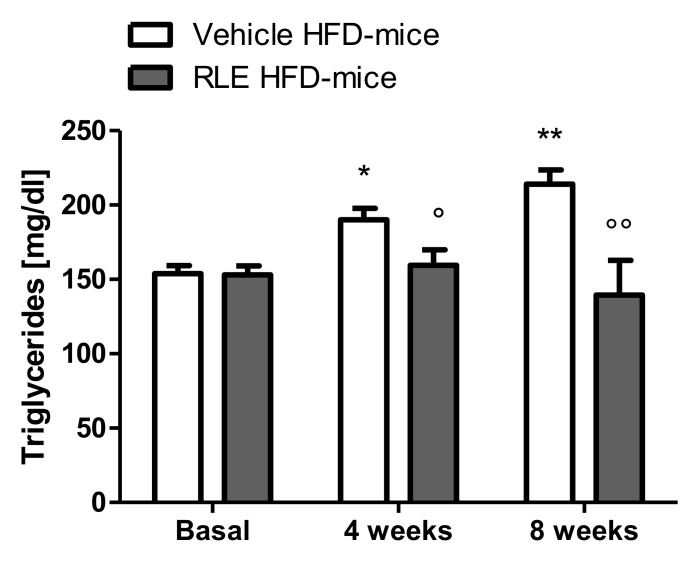
Eight weeks of RLE diet supplementation reduce triglycerides blood concentration in HFD-mice. RLE diet supplementation was able to block blood triglycerides increase in CD1 mice fed on a high-fat diet (HFD-mice) after four and eight weeks of treatment. Data are means ± SEM of 9 to 12 mice per group. Two-way ANOVA followed by Bonferroni post-test. * *p* < 0.5; ** *p* < 0.1 vs vehicle HFD-mice basal group; ° *p* < 0.5; °° *p* < 0.5 vs vehicle HFD-mice 8 weeks group.

**Figure 4:**
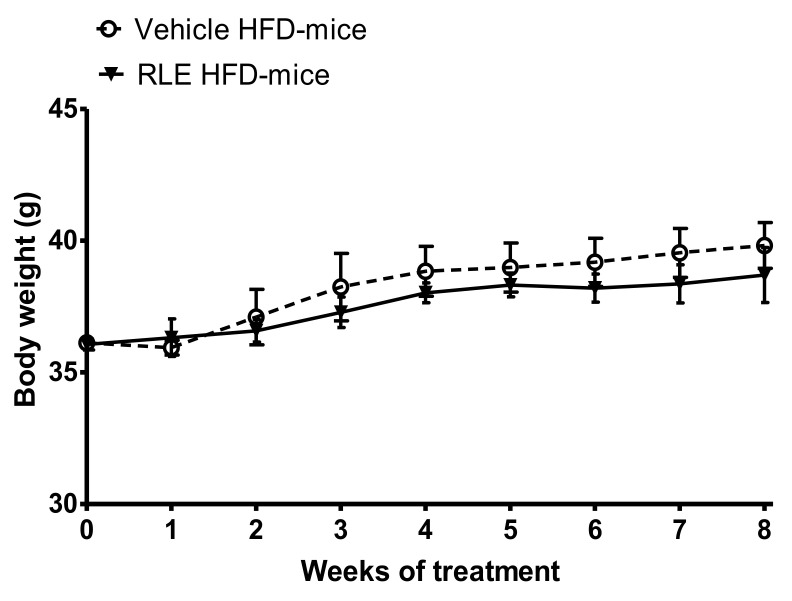
Effect of RLE diet supplementation on body weight increment in HFD-mice. RLE diet supplementation did not significantly reduce body weight increment in HFD-mice over the eight-week treatment. Data are means ± SEM of 9 to 12 mice per group.

In this study, adult male CD1 mice fed with HFD for eight weeks were used to investigate the potential beneficial effect of RLE diet supplementation on the glucose and lipid metabolic asset. Body weight gain was also analyzed over the duration of the study. To assess whether RLE supplementation was able to counteract the negative metabolic effects induced by HFD (i.e., the increase in glucose, cholesterol, and triglycerides blood levels), basal levels of glucose (Figure 1, basal), cholesterol (Figure 2, basal) and triglycerides (Figure 3, basal) were measured before starting any treatment. Mice were then randomly divided into two subgroups, both fed with HFD and treated with either vehicle (Vehicle HFD-mice, white bars) or RLE (RLE HFD-mice, grey bars) for eight consecutive weeks. Glucose, cholesterol, and triglycerides levels were measured after four and eight weeks of treatment. The effects of four and eight weeks of RLE diet supplementation on glucose, as well as cholesterol and triglycerides blood levels in HFD mice, were evaluated and compared to vehicle treatment, while the effects of four and eight weeks of HFD alone were compared to basal levels (Figure 1, Figure 2 and Figure 3, white bars). Results from this study show that RLE supplementation is able to improve glucose and lipid metabolism in mice fed with HFD by preventing the increased blood levels of glucose, cholesterol, and triglycerides. As shown in Figure 1, Figure 2 and Figure 3, four or eight weeks of treatment with RLE was able to maintain all measured parameters within basal ranges. However, RLE supplementation failed to prevent body weight gain in HFD-mice (Figure 4). Although a slight reduction in body weight gain was observed in RLE HFD-mice over the duration of this study, no statistically significant differences were observed between RLE and vehicle groups.

As expected from previous literature data [13], HFD induced an increase in glucose, triglyceride, and cholesterol hematic levels. Specifically, compared to basal levels, eight-week HFD exposure resulted in a significant increase in glucose, cholesterol, and triglyceride blood levels (Figure 1, Figure 2 and Figure 3, white bars). Interestingly, a significant increase in triglycerides was already observed after only four weeks of HFD and became more significant after eight weeks (Figure 3, white bars). In RLE-treated mice, HFD was not able to induce hyperglycemic or hyperlipidemic effects, as all parameters were similar to those measured before HFD (basal). RLE supplementation was able to block HFD-induced triglycerides and cholesterol increases after four weeks of treatment; this effect became more significant after eight weeks of treatment with RLE (Figure 2 and Figure 3, grey bars). In contrast, RLE supplementation did not significantly modify glucose blood levels after four weeks of treatment. However, glucose blood levels were significantly reduced after eight weeks of treatment in RLE HFD-mice compared to vehicle-treated HFD-mice (Figure 1, grey bars).

These results suggest that RLE, which contains anthocyanins and other polyphenols from red orange processing wastes along with eriocitrin and other flavanones from lemon peel, may have beneficial health effects associated with the control of hyperglycemia and hyperlipidemia. Thus, RLE could be proposed as a new phytoextract for nutraceutical purposes.

## 3. Discussion

The encouraging results presented herein corroborate previous findings on different health benefits elicited by RLE. A number of studies have provided evidence of health-promoting properties for many phytochemicals contained in RLE such as flavanones, hydroxycinnamic acids, flavones, and flavonols [7,8,9,10,11,12].

Indeed, it has been previously demonstrated that RLE is active in reducing basophil degranulation and activation [14] and in preventing renal damage induced by diabetic nephropathy [15,16] or ocratoxin ingestion [17]. Moreover, RLE has been previously tested as an oral additive for goat kids, showing ameliorated juiciness and reduced color deterioration of the kids’ meat as a result of delayed lipid oxidation. This resulted in an improved fatty acid profile and thus healthier meat for human consumption [18]. It has also been previously demonstrated that goat kids fed on a diet supplemented with RLE showed significantly increased neuropeptide Y-(a neuropeptide involved in food intake regulation) immunoreactive cells in the abomasal epithelium and pancreatic islets, representing a baseline for future studies on the interaction between neuropeptides and polyphenols [19].

Dietary fat intake is known to be a major risk factor for hyperlipidemia and hyperglycemia, causing severe metabolic disturbances [20]. Elevated plasma lipids, mainly cholesterol and triglycerides, are important risk factors for a number of cardiovascular diseases including atherosclerosis, stroke, and hyperglycemia, as well as for the development of diabetes [20]. Dietary fat intake in mice is known to induce similar metabolic changes to those observed in humans, providing a suitable model for the study of HFD-induced metabolic diseases [13] as well as for the study of potentially active compounds [21]. In the present study, we demonstrated that eight weeks of RLE supplementation prevented HFD-induced hyperlipidemia and hyperglycemia by reducing triglycerides, total cholesterol, and glucose blood levels in mice. Although RLE was not able to significantly reduce body weight gain in HFD-mice at the dose tested and for the duration of the study, results obtained on glucose and lipid blood levels encourage to further investigate biochemical mechanisms that underlie these effects.

Further studies will be aimed at investigating whether RLE treatment is able to affect specific intestinal transporters such as the transmembrane protein CD36, and the glucose transporters SGLT1 and GLUT2. CD36 is known to be expressed in many cell types including enterocytes [22,23,24] and to finely regulate lipid uptake [25,26]. Furthermore, SGLT1 and GLUT2 are known to play an important role in intestinal glucose transport [27]. Interestingly enough, dietary anthocyanins and polyphenols are known to regulate CD36 gene expression [28], as well as to interfere with SGLT1 and GLUT2 transporters [29].

Altogether, these results support the role of phytochemicals such as anthocyanins, polyphenols, eriocitrin, and other flavanones in preventing some of the most dangerous effects of excessive dietary fat intake and provide evidence for the benefits of supplementation of RLE in the prevention of metabolic diseases induced by HFD.

## 4. Materials and Methods

### 4.1. Chemicals and Reagents

Standardized red orange and lemon extract (RLE) was obtained with a patented extraction process from blood orange and lemon processing wastes in the CREA lab (Italian Patent No 102017000057761 “Metodo per la produzione di un estratto da sottoprodotti della lavorazione degli agrumi ed estratto così ottenuto” owned by CREA). MultiCare in reactive strips (Biochemical Systems International) were used to measure glucose, total cholesterol, and triglycerides hematic levels.

### 4.2. Chemical Composition of RLE

Anthocyanin components (g/100 g of powder extract) were determined by HPLC-PDA-ESI/MSn according to a previously published method [14] using an ultra-fast HPLC system coupled to a photodiode array (PDA) detector and Finnigan LXQ ion trap equipped with an electrospray ionization (ESI) interface in series configuration (Thermo Electron, San Jose, CA, USA). RLE was diluted in water for HPLC analysis of anthocyanins and loaded onto a C18 SPE cartridge for purification. Recovered pigments were redissolved in 7% aqueous formic acid and subjected to analysis. Separation of anthocyanins was conducted on a Chromolith Performance RP-18 end-capped column (100 × 3.0 mm i.d., monolithic particle size; Merck KGaA, Darmstadt, Germany), and a binary gradient of water with 7% formic acid and methanol was used as eluent. The flow rate was 300 μL/min. The column effluent was monitored at 520 nm. The calibration curve for anthocyanin compounds quantification was y = 265595x obtained using Cya-3-glu as pure standard. The HPLC chromatogram is reported in Figure S1. Flavanone glycosides, expressed as hesperidin equivalents (g/100 g of powder extract), were determined by using the HPLC-PDA-ESI/MSn equipment described above, based on a previously published method [14]. Briefly, RLE was dissolved in dimethyl sulfoxide and further diluted with the mobile phase (water:acetonitrile:acetic acid = 79.5:20:0.5). The solution was filtered through a 0.45 μM membrane filter and then injected directly into the HPLC system. The column was the same employed for anthocyanins analysis and the flow rate was 800 μL/min. Detection was monitored at 280 nm. The calibration curve for flavanone compounds quantification was y = 83099x using hesperidin as the pure standard. The HPLC chromatogram is reported in Figure S1. Hydroxycinnamic acids (p-coumaric, ferulic, and sinapic acids) were quantified and expressed as g/100 g of powder extract according to the published methods [12,30]. Hydroxycinnamates were determined after alkaline hydrolysis of hydroxycinnamic esters followed by acidification and further purification through solid-phase extraction (SPE). The eluents used for HPLC analysis were water/acetic-acid (98:2) (solvent A) and methanol (solvent B) with a gradient transition from 95% to 70% of solvent A over 35 min. Separation was conducted on an Onyx Monolithic C18 column (100 × 3.0 mm i.d., monolithic particle size; Phenomenex, Torrance, CA, USA). The flow rate was 1 mL/min, and the detection was performed at 310 nm. The calibration curve for hydroxycinnamic acids quantification was y = 143810x for *p*-coumaric acid, y = 85743x for ferulic acid, and y = 62206x for sinapic acid. The HPLC chromatogram is reported in Figure S1.

### 4.3. In Vivo Studies

Animals. For the evaluation of the biological activity of RLE, 9 to 12 CD1 male mice per experimental group, aged between 6 and 7 weeks, were used. All experiments were carried out in accordance with experimental protocols approved by the Italian Ministry of Health.

RLE administration. RLE was solubilized in water and mice received a daily oral administration of RLE containing 120 mg/kg/day^−1^ of total anthocyanins. The main individual compounds were cyanidin 3-glucoside, counting for 50 mg/kg/day^−1^, and cyanidin 3-(6-malonyl) glucoside), counting for 25 mg/kg/day^−1^. The total intake of flavanones amounted to 700 mg/kg/day^−1^ with the main individual compounds being eriocitrin (480 mg/kg/day^−1)^, narirutin (60 mg/kg/day^−1^), hesperidin (150 mg/kg/day^−1^), and didymin (10 mg/kg/day^−1^).

High-fat diet. An adjusted calories diet was used for this study (Teklad Custom Research Diet, TD.06414) containing 60.3% of total calories from fat, 18.4% from proteins, and 21.3% from carbohydrates.

Evaluation of blood glucose, cholesterol, and triglycerides levels. Mice were fed on a high-fat diet (Harlan-DIETA TD 06414) for eight weeks and randomly divided into two groups. Concomitantly to the high-fat diet, one group received the RLE supplement (RLE-HFD mice) while the control group received water (Vehicle-HFD mice). For the evaluation of glucose, cholesterol, and triglycerides levels, 100 microliters of blood were drawn from the tail vein and a drop was put on multiCare in reactive strips (Biochemical Systems International). Results were expressed as mg/dl. Glucose, cholesterol, and triglycerides levels were evaluated before the high-fat diet administration (basal) or treatments and after 4 and 8 weeks of treatment. Glucose, cholesterol, and triglycerides blood levels were measured in mice before any treatment (basal) and after four and eight weeks of HFD.

### 4.4. Statistical Analysis

Data are the mean ± SEM of 9 to 12 mice per group. Statistical significance was determined using a two-way ANOVA followed by the Bonferroni post-test. In all cases, *p* < 0.05 was considered statistically significant.

## Figures and Tables

**Table 1:** Identification and relative amounts of anthocyanins in RLE.

Compound No.	[M]^+^ (*m*/*z*)	MS*^n^* (*m*/*z*)	Anthocyanin	Relative Composition ^a^ (%)
1	611	449/287	cyanidin 3,5-diglucoside	1.19
2	465	303	delphinidin 3-glucoside	2.69
3	611	287	cyanidin 3-sophoroside	0.39
4	449	287	cyanidin 3-glucoside	39.94
5	595	287	cyanidin 3-rutinoside	1.32
6	479	317	petunidin 3-glucoside	1.47
7	551	465/303	delphinidin 3-(6″-malonyl)glucoside	1.41
8	463	301	peonidin 3-glucoside	2.93
9	565	479/317	petunidin 3-(6″-malonyl)glucoside	1.48
10	535	449/287	cyanidin 3-(6″-malonyl)glucoside	21.86
11	593	449/287	cyanidin 3-(6″-dioxalyl)glucoside	5.70
12	-	271	pelargonidin derivative	1.34
13	549	463/301	peonidin 3-(6″-malonyl)glucoside	13.85
14	-	287	cyanidin derivative	2.29
15	-	301	peonidin derivative	1.85
Total anthocyanins (g Cya-3-glu equivalents/100 g) ^b^				2.56 ± 0.08

^a^ Relative content of anthocyanins calculated from peak areas recorded at 520 nm. ^b^ Results are expressed as the mean ± standard deviation (n = 3).

**Table 2:** Identification and concentration values of flavanones in RLE.

Compound No.	[M‒H]^‒^ (*m*/*z*)	MS*^n^* (*m*/*z*)	Flavanone	g/100 g ^a^
1	595	287	eriocitrin	11.02 ± 0.02
2	579	271	narirutin	1.23 ± 0.01
3	609	301	hesperidin	3.46 ± 0.01
4	593	285	didymin	0.15 ± 0.02
Total flavanones (g Hesperidin equivalents/100 g)				15.86 ± 0.01

^a^ Results are expressed as the mean ± standard deviation (n = 3).

**Table 3:** Identification and concentration values of hydroxycinnamic acids in RLE.

Compound No.	[M‒H]^‒^ (*m*/*z*)	MS*^n^* (*m*/*z*)	Hydroxycinnamic Acids	g/100 g ^a^
1	325	163/145	*p*-coumaric	0.366 ± 4.13
2	355	193/175	ferulic	0.970.± 3.72
3	385	223/205	sinapic	0.432 ± 1.45
Total hydroxycinnamic acids (g/100 g)				1.77 ± 0.009

^a^ Results are expressed as the mean ± standard deviation (n = 3).

## Data Availability

Data available from the authors.

## Supplementary Materials

The following are available online. Figure S1: HPLC-PDA chromatograms detected at (A) 520 nm for anthocyanin compounds; (B) 280 nm for flavanones compounds and (C) 310 nm for hydroxycinnamic acids.

## Sample Availability

Samples of the compounds are available from the authors.

## References

[1] Food and Agriculture Organization of the United Nations Database. http://www.fao.org/faostat/en/.

[2] Rapisarda P., Tomaino A., Cascio R.L., Bonina F., de Pasquale A., Saija A. (1999). Antioxidant Effectiveness As Influenced by Phenolic Content of Fresh Orange Juices. J. Agric. Food Chem..

[3] Castañeda-Ovando A., Pacheco-Hernández M.D., Páez-Hernández M.E., Rodríguez J.A., Andrés C. (2009). Galán-Vidal Chemical studies of anthocyanins: A review. Food Chem..

[4] Titta L., Trinei M., Stendardo M., Berniakovich I., Petroni K., Tonelli C., Riso P., Porrini M., Minucci S., Pelicci P.G. (2010). Blood orange juice inhibits fat accumulation in mice. Int. J. Obes..

[5] Rapisarda P., Bellomo S., Intrigliolo F. (2000). Recent research developments in agricultural & food chemistry. Res. Signpost.

[6] Saija A., Tomaino A., Cascio R.L., Rapisarda P., Dederen J.C. (1998). In vitro antioxidant activity and in vivo photoprotective effect of a red orange extract. Int. J. Cosmet. Sci..

[7] Emim J.A.D.S., Oliveira A.B., Lapa A.J. (1994). Pharmacological Evaluation of the Anti-inflammatory Activity of a Citrus Bioflavonoid, Hesperidin, and the Isoflavonoids, Duartin and Claussequinone, in Rats and Mice. J. Pharm. Pharmacol..

[8] González-Molina E., Domínguez-Perles R., Moreno D.A., García-Viguera C. (2010). Natural bioactive compounds of *Citrus limon* for food and health. J. Pharm. Biomed. Anal..

[9] Miyake Y., Yamamotoi K., Osawa T. (1997). Isolation of eriocitrin (eriodictyol 7-rutinoside) from lemon fruit (*Citrus limon* BURM. f.) and its antioxidative activity. Food Sci. Technol. Int. Tokyo.

[10] Miyake Y., Yamamoto K., Morimitsu Y., Osawa T. (1997). Isolation of C-Glucosylflavone from Lemon Peel and Antioxidative Activity of Flavonoid Compounds in Lemon Fruit. J. Agric. Food Chem..

[11] Del Río J.A., Fuster M.D., Gómez P., Porras I., García-Lidón A., Ortuño A. (2004). *Citrus limon*: A source of flavonoids of pharmaceutical interest. Food Chem..

[12] Amenta M., Ballistreri G., Fabroni S., Romeo F.V., Spina A., Rapisarda P. (2015). Qualitative and nutraceutical aspects of lemon fruits grown on the mountainsides of the Mount Etna: A first step for a protected designation of origin or protected geographical indication application of the brand name ‘Limone dell’Etna’. Food Res. Int..

[13] Li J., Wu H., Liu Y., Yang L. (2020). High fat diet induced obesity model using four strainsof mice: Kunming, C57BL/6, BALB/c and ICR. Exp. Anim..

[14] Caruso M., Fabroni S., Emma R., Ballistreri G., Amenta M., Currenti W., Rinzivillo C., Rapisarda P. A new standardized phytoextract from red orange and lemon wastes (red orange and lemon extract) reduces basophil degranulation and activation. Nat. Prod. Res..

[15] Damiano S., Lauritano C., Longobardi C., Andretta E., Elagoz A.M., Rapisarda P., Di Iorio M., Florio S., Ciarcia R. (2020). Effects of a Red Orange and Lemon Extract in Obese Diabetic Zucker Rats: Role of Nicotinamide Adenine Dinucleotide Phosphate Oxidase. J. Clin. Med..

[16] Damiano S., Lombari P., Salvi E., Papale M., Giordano A., Amenta M., Ballistreri G., Fabroni S., Rapisarda P., Capasso G. (2019). A red orange and lemon by-products extract rich in anthocyanins inhibits the progression of diabetic nephropathy. J. Cell. Physiol..

[17] Damiano S., Iovane V., Squillacioti C., Mirabella N., Prisco F., Ariano A., Amenta M., Giordano A., Florio S., Ciarcia R. (2020). Red orange and lemon extract prevents the renal toxicity induced by ochratoxin A in rats. J. Cell. Physiol..

[18] Salzano A., Damiano S., D’Angelo L., Ballistreri G., Claps S., Rufrano D., Maggiolino A., Neglia G., De Palo P., Ciarcia R. (2021). Productive Performance and Meat Characteristics of Kids Fed a Red Orange and Lemon Extract. Animals.

[19] De Felice E., Giaquinto D., Damiano S., Salzano A., Fabroni S., Ciarcia R., Scocco P., de Girolamo P., D’angelo L. (2021). Distinct pattern of npy in gastro–entero–pancreatic system of goat kids fed with a new standardized red orange and lemon extract (Rle). Animals.

[20] O’Keefe J.H., Bell D.S. (2007). Postprandial hyperglycemia/hyperlipidemia (postprandial dysmetabolism) is a cardiovascular risk factor. Am. J. Cardiol..

[21] Lee H.S., Nam Y., Chung Y.H., Kim H.R., Park E.S., Chung S.J., Kim J.H., Sohn U.D., Kim H.C., Oh K.W. (2014). Beneficial effects of phosphatidylcholine on high-fat diet-induced obesity, hyperlipidemia and fatty liver in mice. Life Sci..

[22] Poirier H., Degrace P., Niot I., Bernard A., Besnard P. (1996). Localization and regulation of the putative membrane fatty-acid transporter (FAT) in the small intestine. Comparison with fatty acid-binding proteins (FABP). Eur. J. Biochem..

[23] Chen M., Yang Y., Braunstein E., Georgeson K.E., Harmon C.M. (2001). Gut expression and regulation of FAT/CD36: Possible role in fatty acid transport in rat enterocytes. Am. J. Physiol. Endocrinol. Metab..

[24] Vega M.A., Bragado R. (2001). Localization of the lipid receptors CD36 and CLA-1/SR-BI in the human gastrointestinal tract: Towards the identification of receptors mediating the intestinal absorption of dietary lipids. J. Histochem. Cytochem..

[25] Goudriaan J.R., den Boer M.A., Rensen P.C., Febbraio M., Kuipers F., Romijn J.A., Havekes L.M., Voshol P.J. (2005). CD36 deficiency in mice impairs lipoprotein lipase-mediated triglyceride clearance. J. Lipid Res..

[26] Werder M., Han C.H., Wehrli E., Bimmler D., Schulthess G., Hauser H. (2001). Role of scavenger receptors SR-BI and CD36 in selective sterol uptake in the small intestine. Biochemistry.

[27] Röder P.V., Geillinger K.E., Zietek T.S., Thorens B., Koepsell H., Daniel H. (2014). The role of SGLT1 and GLUT2 in intestinal glucose transport and sensing. PLoS ONE.

[28] Kao E.S., Tseng T.H., Lee H.J., Chan K.C., Wang C.J. (2009). Anthocyanin extracted from Hibiscus attenuate oxidized LDL-mediated foam cell formation involving regulation of CD36 gene. Chem. Biol. Interact..

[29] Solverson P. (2020). Anthocyanin Bioactivity in Obesity and Diabetes: The Essential Role of Glucose Transporters in the Gut and Periphery. Cells.

[30] Rapisarda P., Lo Bianco M., Pannuzzo P., Timpanaro N. (2008). Effect of cold storage on vitamin C, phenolics and antioxidant activity of five orange genotypes [*Citrus sinensis* (L.) Osbeck]. Postharvest Biol. Technol..

