# Supplementary material for: A Standardized Extract Prepared from Red Orange and Lemon Wastes Blocks High-Fat Diet-Induced Hyperglycemia and Hyperlipidemia in Mice
Source: Molecules. 2021 Jul 15;26(14):4291. doi: 10.3390/molecules26144291 (PMC8304280; doi:10.3390/molecules26144291)
Supplement: Supplementary file 1 [file molecules-26-04291-s001.zip › molecules-1259012-supplementary.pdf]

Supplementary Material

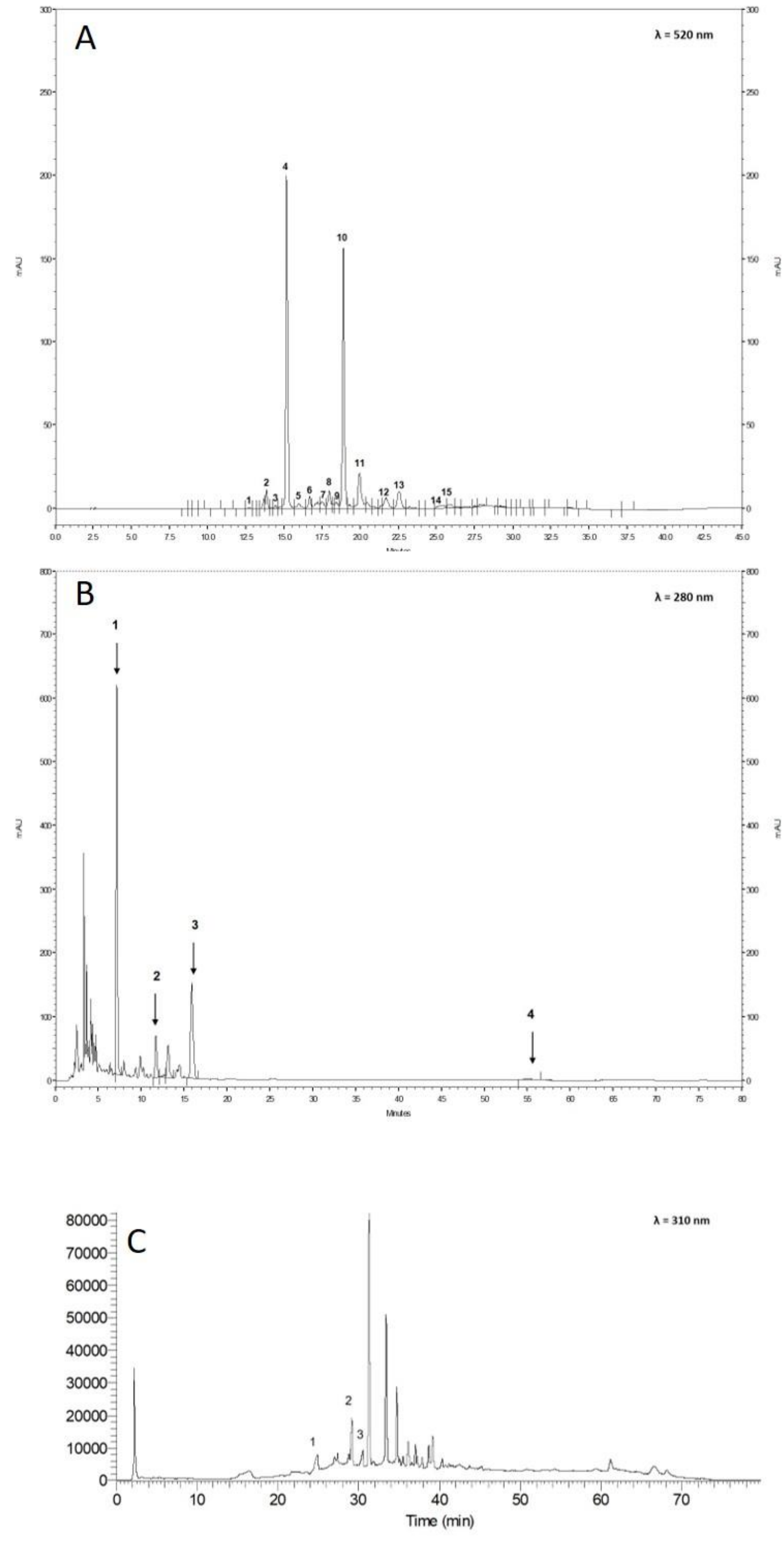

Figure S1 HPLC-PDA chromatograms detected at A) 520 nm for anthocyanin compounds; B) 280 nm for flavanones compounds and C) 310 nm for hydroxycinnamic acids.
